# Plant-Derived Exosome-like Nanoparticles: Biocompatible Vectors for Efficient Intervention of Autoimmune Diseases

**DOI:** 10.3390/ijms27219866

**Published:** 2026-11-03

**Authors:** Lingyan Zhao, Boyang Hu, Zhenmeng Ren, Ke Li, Yuxin Ma, Lu Wang, Yang Liu

**Affiliations:** College of Basic Medical Sciences, Shanxi University of Chinese Medicine, Jinzhong 030619, China; 15252606940@139.com (L.Z.); 18237834233@163.com (B.H.); 13935994273@163.com (Z.R.); like77722@163.com (K.L.); myx1902168364@163.com (Y.M.)

**Keywords:** plant-derived exosome-like nanoparticles, autoimmune diseases, biological characteristics of PELNs, targeted regulation, engineering, safety

## Abstract

Autoimmune diseases (AIDs), a group of diseases induced by dysfunction of the immune system, are currently prompting the search for novel intervention strategies. Recent studies have found that plant-derived exosome-like nanoparticles (PELNs) have the potential to restore immune homeostasis. In particular, preclinical evidence indicates that PELNs can modulate the immune microenvironment, inhibit inflammatory pathways, and exert antioxidant and tissue-repair effects. Meanwhile, molecular loading, targeted modification and novel engineering strategies have significantly improved the Intervention precision of PELNs. Based on this, this review summarizes the biological functions, functional advantages, and regulatory mechanisms of PELNs in AIDs and discusses the prospects for their large-scale clinical application in terms of engineering modification, preparation standardization, and multidimensional safety assessment, with the aim of providing a theoretical basis and reference for the development of novel intervention strategies for AIDs.

## 1. Introduction

Autoimmune diseases (AIDs) are chronic inflammatory disorders caused by immune system dysfunction, in which the immune system mistakenly attacks the body’s normal tissues and organs. Statistics indicate that their incidence in the general population worldwide is approximately 3%–5%. These diseases are characterized by insidious onset, a prolonged course, and frequent recurrence, imposing long-term physical and psychological burdens on patients [1]. Currently, treatment of AIDs relies primarily on nonspecific immunosuppressants, such as glucocorticoids [2]. However, prolonged use may substantially increase the risk of infection. Therefore, novel therapeutic strategies that effectively regulate immune responses while reducing adverse effects are urgently needed to improve the long-term quality of life of patients with AIDs [3].

Recent studies have shown that plant-derived exosome-like nanoparticles (PELNs) possess unique advantages, including good biocompatibility and ease of engineering modification, and they can participate in regulating immune cell balance, inhibiting key inflammatory signaling pathways, and promoting the reconstruction of immune tolerance. These findings provide insights into the development of effective and safe intervention strategies for AIDs [4]. However, existing reviews have mainly focused on the immunoregulatory mechanisms of PELNs, without comprehensively exploring their current research status and prospects in AIDs. Accordingly, this review examines three aspects: the active components of PELNs, the specific mechanisms underlying PELN-mediated intervention in AIDs, and strategies for their clinical translation. It also summarizes potential opportunities and challenges in clinical translation from the perspectives of engineering, standardization, and safety, providing a theoretical reference for the development of next-generation PELN-based intervention agents for AIDs.

## 2. Formation of Plant-Derived Vesicles and Bioactive Substances

### 2.1. PELNs/PDEVs Represent Two Major Categories of Plant-Derived Vesicles

Terminology in the field of plant-derived nanovesicles has not yet been fully standardized. Based on existing studies and the Minimal Information for Studies of Extracellular Vesicles 2018 (MISEV2018) guidelines [5], these vesicles can be classified as follows: (1) Plant-derived extracellular vesicles (PDEVs), also termed plant extracellular vesicles (PEVs), are vesicles naturally and actively secreted by living plant cells and isolated from apoplastic washing fluid. (2) Plant-derived vesicle-like nanoparticles (PDVLNs), plant-derived nanovesicles (PDNVs), and plant-derived exosome-like nanoparticles (PELNs) refer to heterogeneous preparations obtained by mechanically disrupting plant tissues through grinding, juicing, homogenization, or related methods. These preparations contain naturally secreted vesicles, intracellular membrane fragments, and organelle fragments [6]. Since the evidence that plant multivesicular bodies (MVBs) release PDEVs remains insufficient, plant-derived samples cannot be equated with mammalian exosomes (M-Exos) and similarly termed exosomes. Although these terms are frequently used interchangeably, they represent fundamentally different things [5] (Table 1, Supplementary Table S1).

### 2.2. The Formation of PDEVs Is a Key Mechanism by Which Plant Cells Respond to Environmental Stress and Maintain Immune Homeostasis

Although both PDEVs and PELNs mediate immune regulation, they originate from different sources. Therefore, this article clearly distinguishes between them in terms of their formation. PDEVs are present in the extracellular space and represent intercellular communication and material transport. The most widely reported secretion pathway for PDEVs is the multivesicular body (MVB) pathway [7].

MVBs are late endosomes containing intraluminal vesicles (ILVs), also referred to as prevacuolar compartments (PVCs). MVBs generally have two possible fates: (1) Fusion with the plasma membrane, releasing their ILVs into the extracellular space. (2) Fusion with vacuoles or lysosomes for degradation, facilitating the clearance and recycling of intracellular components [8,9].

The MVB-mediated extracellular vesicle (EV) biogenesis pathway was first reported in plants, and multiple lines of evidence support the existence of an MVB-mediated vesicle secretion pathway in plants [10]. In 1965, JENSEN first observed EVs outside cotton cells using transmission electron microscopy [11]. Subsequently, fusion of MVBs with the plasma membrane (PM) was reported in a carrot cell culture system [12]. Early studies of endocytosis in soybean protoplasts showed that extracellular vesicles were attached to the plasma membrane. Moreover, identical molecular components, such as arabinogalactan proteins and peroxidases, exhibited immunocolocalization in MVBs and paramural bodies, indicating that some vesicles within paramural bodies originate from MVBs [13].

Both PDEVs and mammalian exosomes (M-Exos) share an MVB biogenesis pathway, but the evidence supporting this pathway differs between them. In mammalian systems, the fusion of MVBs with the plasma membrane and the release of ILVs to generate exosomes (Exos) have been confirmed by multiple lines of evidence, including in vivo fluorescence tracking and molecular mechanisms [14,15]. When plant cells encounter stress, such as pathogen infection, MVB structures (paramural bodies) can be observed in the space between the plasma membrane and the cell wall. Their formation has mainly been characterized through static ultrastructural observations using transmission electron microscopy and fluorescence immunocolocalization. However, direct dynamic evidence of MVB–PM fusion has not yet been obtained in plants [16].

Specific proteins guide PDEVs maturation and cargo sorting. Some studies have proposed that the endosomal sorting complex required for transport (ESCRT) is one of the mediating components. TOM1-like (TOL) proteins perform ubiquitin recognition and sorting functions in place of ESCRT-0 through their conserved VHS domains and participate in material transport in early endosomes [17]. The ESCRT-I, ESCRT-II, and ESCRT-III complexes participate in cargo sorting, membrane invagination, and ILV budding. Notably, although ESCRT is associated with PDEV generation in Arabidopsis cell culture systems, the current evidence demonstrates only a correlation at the transcriptional level rather than direct causal evidence [18].

Endoplasmic reticulum membrane-localized Ras-related protein Rab-1/Ras-related protein Rab-D (Rab1/RabD) mediates the directional transport of early sorting endosomes (ESEs) to the Golgi apparatus by recruiting specific effector proteins. In contrast, Golgi membrane-localized Ras-related protein Rab-2A/RAB GTPase homolog A2B (Rab2/RabB) is responsible for the transport of ESEs within the Golgi apparatus and the recycling of ILVs in the cytoplasm [19]. In addition, the plant trans-Golgi network (TGN) simultaneously performs endocytosis and sorting under the regulation of Ras-related GTP-binding proteins (Rab GTPases) [20]. Under the regulation of VPS9A (Rab guanine nucleotide exchange factor), the Ras-related protein Rab-5 homologs Ras-related protein RABF2a/Ras-related protein Rha1 (RabF2a/RHA1) are responsible for delivering early endosomes (EEs) to the TGN/EE. Ras-related protein RABF2b/Arabidopsis Rab GTPase homolog F2b (RabF2b/ARA7) mediates the maturation of multivesicular bodies (MVBs) [21]. The plant-specific Ras-related protein RABF1/*Arabidopsis thaliana* Ras-related GTP-binding protein (RabF1/ARA6) undergoes lipid modification at its N-terminus and participates in vesicle degradation and recycling. Studies have shown that this sorting mechanism helps enhance the innate immune capacity of plants against fungal pathogens [22].

A plant-specific non-classical secretion pathway involving exocyst-positive organelles (EXPOs) also exists [23]. Evidence from vesicle membrane and peripheral markers indicates that Exo70E2 is released following vesicle rupture and directly delivers defense proteins to the extracellular space, accelerating their transport to pathogen infection sites [24]. Furthermore, during MVB secretion, plants can transiently loosen cell wall structures by locally secreting cell wall remodeling factors, such as expansins, pectinases, and hydrolases, thereby providing channels for PELN release [10,23] (Figure 1).

### 2.3. An Efficacy-Oriented Research Strategy for PELNs

PELNs are generated during the mechanical disruption of plant tissues and represent a heterogeneous mixture of vesicles and non-vesicular nanoparticles rather than clearly defined secreted extracellular vesicles [25]. Their cargo depends on the extraction and purification methods used. In studies investigating the biological mechanisms of PELNs, researchers generally do not strictly distinguish PDEVs from PELNs. Instead, they evaluate centrifugation-derived PELNs as an integrated functional preparation. For example, GELENs were obtained by sucrose-gradient centrifugation from two interfaces, 8%–30% and 30%–45%. Although the fractions differed in mass and lipid content, the investigators emphasized their ability to mediate Nrf2 activation and hepatic ROS production rather than determining the extracellular origin of individual particles [26]. Similarly, studies of broccoli-derived exosome-like nanoparticles (B-ELNs) have focused on a mechanistic framework involving integrated transcriptomic and proteomic reprogramming, emphasizing multitarget regulation rather than attributing the effects to a single vesicle subpopulation [27]. Thus, current PELN research reflects a pragmatic, efficacy-oriented approach, prioritizing functional mechanisms over improvements in PELNs purity alone.

### 2.4. Bioactive Substances Enriched in PELNs Are the Material Basis for Their Immunomodulatory Functions

#### 2.4.1. Lipids

Lipidomic analysis has shown that, compared with M-Exos, PELNs have a more diverse phospholipid composition, including glycerophospholipids (GPLs), such as phosphatidylcholines (PCs), phosphatidylethanolamines (PEs), phosphatidylinositols (PIs), phosphatidylserines (PSs), and phosphatidic acid (PA), as well as phytosterols [28]. These unique lipid components collectively confer superior membrane stability and structural integrity on PELNs, providing a broader range of functional options for intervening in AIDs.

First, phytosterols contained in PELNs, such as β-sitosterol, can reduce membrane fluidity and enhance membrane compactness. For example, when β-sitosterol is incorporated into plant phospholipid (DLiPC) membranes at a proportion of 50 mol%, the disordered liquid phospholipid membrane transitions to an ordered state [29]. Second, PC has a stable connection between its glycerol backbone, fatty acids, and choline group, which may contribute to PELN stability [30]. Studies have shown that, compared with liposomes, the ordered arrangement of phospholipids, including PC, in AELNs prevents Triton X-100 from inserting into the lipid bilayer, allowing AELNs to maintain their particulate morphology in Triton X-100 [31]. In addition, the negatively charged head group of PA is located close to the alkyl chain region and has an affinity for divalent cations. It may regulate membrane fission and fusion through the formation of intermolecular hydrogen bonds, thereby significantly improving PELN stability [32,33,34]. Therefore, we believe that the composition of multiple lipids can enhance the structural integrity of PELNs in complex physiological environments.

The phospholipid composition of PELNs is not fixed, and their lipid content and related functions vary significantly depending on the plant species. For example, although *Curcuma longa*-derived exosome-like nanoparticles (CLEs) and ginger-derived exosome-like nanoparticles (GELNs), both belonging to the Zingiberaceae family, are extracted from rhizomes, the PA content of GELNs is higher than that of CLEs [35,36].

#### 2.4.2. Proteins

The proteins contained in PELNs are mainly cytoplasmic proteins, including actin, protein kinases, channel proteins, and annexins, which collectively form the structural framework of PELNs and participate in their transport functions [37]. Protein kinases and G proteins, as key molecules in signal transduction, mediate the involvement of PELNs in intercellular communication. Intracellular transport proteins, such as channel proteins and annexins, are responsible for the transport and delivery of PELN contents [38]. Proteins such as ubiquitin, clathrin, and ALIX are core sorting factors that regulate PELN biogenesis [39]. Members of the integrin family, such as endoplasin, mediate specific binding between PELNs and receptors on the surfaces of target cells, thereby initiating downstream biological effects [40].

Currently, the more widely recognized markers include Penetration 1 (PEN1), Tetraspanin-8 (TET-8), and Tetraspanin-9 (TET9) [41]. PEN1 is a plastid-localized, Mn^2+^-dependent 5′–3′ exonuclease [42]. Under pathogen stress, PEN1 is secreted into the extracellular matrix of the cell wall and participates in PELN-mediated immune responses. Fluorescence tracking signals for TET8/TET9 have been detected in MVBs [43], and subsequent genetic experiments confirmed that MVBs carrying TET8/TET9 can transport functional RNA and are actively involved in PELN-mediated immune regulation [44]. Meanwhile, classical mammalian markers, including CD9, CD63, CD81, and TSG101, have also been detected in PELNs, but the protein characteristics of these markers require more detailed investigation [45,46]. Increasing evidence indicates that PELN identification should integrate multiple indicators, including cargo composition and biological activity, rather than relying solely on conserved proteins as a single criterion [47].

#### 2.4.3. Non-Coding RNA

Non-coding RNA (ncRNA) refers to a family of RNA molecules that do not encode proteins, including microRNA (miRNA) and small interfering RNA (siRNA) [48]. These molecules are selectively packaged into EVs and delivered to recipient cells, where they inhibit translation or mediate mRNA degradation, thereby achieving precise regulation of protein-coding genes [49].

The biogenesis processes of animal-derived miRNAs and plant-derived miRNAs (p-miRNAs) differ [50,51]. Mechanistically, p-miRNAs bypass the Drosha-dependent initial processing of animal primary microRNAs (pri-miRNAs) and the stepwise nuclear and cytoplasmic processing of precursor microRNAs (pre-miRNAs). Instead, two cleavage steps are directly completed by the Dicer-like 1 (DCL1) complex in the nucleus. This cleavage pattern results in a more conserved length range for mature plant-derived miRNAs [52,53]. Plant-derived microRNA (p-miRNA) duplexes undergo 2′-O-methylation at their 3′ ends by the methyltransferase Hua Enhancer 1 (HEN1), which significantly prevents the degradation of miRNA duplexes by nucleases [54]. This is also a reason why PELNs can serve as stable delivery carriers. In terms of structure and modification, p-miRNAs can effectively resist exonuclease-mediated degradation, allowing them to maintain greater integrity and a longer half-life in complex biological environments [55].

The sorting mechanism of siRNA has been experimentally demonstrated. In Arabidopsis PELNs, AGO1 and RNA helicases (RH11 and RH37) selectively package siRNAs and deliver them to genes associated with fungal pathogenicity, positively regulating plant immunity against *Pseudomonas syringae* [38]. In summary, substantial research has investigated the ncRNA-mediated the cross-kingdom regulatory effects of PELNs, which is also why PELNs have become a key research focus for cross-kingdom regulation of inflammation-, immunity-, and apoptosis-related pathways.

#### 2.4.4. Secondary Metabolites

During their formation, PELNs selectively package various secondary metabolites with anti-inflammatory and antioxidant activities, including flavonoids, saponins, polysaccharides, and alkaloids [56]. Among the substances detected in PELNs are 6-gingerol from ginger, curcumin from turmeric, and ginsenoside Rg3 from ginseng [57,58]. These small molecules mainly accumulate passively within vesicle membranes through hydrophobic interactions and can target key proteins in the inflammatory, oxidative stress, and apoptosis signaling pathways in animal cells [59]. Currently, the main mechanisms underlying the cross-species effects of secondary metabolites include the following:

(1) Competitive inhibition: Secondary metabolites can directly enter the active site of a target enzyme and compete with endogenous substrates for the same binding site. For example, flavonoids bind to the adenosine triphosphate (ATP)-binding pocket of Janus kinase (JAK), competitively inhibiting ATP-mediated phosphorylation of signal transducer and activator of transcription 1 (STAT1), thereby blocking interferon responses and the initiation of downstream immune responses [60,61].

(2) Allosteric regulation: Secondary metabolites can bind to allosteric sites on target proteins and indirectly regulate their activity by inducing conformational changes. For example, the natural polyphenol resveratrol enhances the deacetylase activity of SIRT1 by altering its conformation. Activated SIRT1 deacetylates transcription factors such as nuclear factor kappa-B (NF-κB) and p53, thereby alleviating inflammatory responses and apoptosis [62].

(3) Interference with protein interactions: Some secondary metabolites can block protein interactions through covalent or non-covalent mechanisms [63]. For example, the diterpenoid oridonin can covalently modify the Cys279 site of the NOD-like receptor family pyrin domain containing 3 (NLRP3) protein, blocking its interaction with NIMA-related kinase 7 (NEK7) and inhibiting the assembly and activation of the NLRP3 inflammasome. In addition, flavonoids can inhibit NLRP3 oligomerization, the activation of cysteine-aspartic acid protease 1 (caspase-1), and alleviate pyroptosis [64] (Table 2).

## 3. Applications of PELNs in Autoimmune Diseases

Currently, research on PELNs in AIDs is still mainly based on animal experiments, and clinical data remain relatively limited [65]. Nevertheless, PELNs have demonstrated potential application value in regulating immune cell functions, inhibiting inflammatory signaling pathways, and improving the tissue microenvironment.

### 3.1. Rheumatoid Arthritis

Rheumatoid arthritis (RA) is characterized by chronic inflammation of the joints and the whole body, pannus formation, and cartilage destruction [66]. This inflammation originates from long-term stimulation of the synovium by genetic susceptibility and environmental factors, which subsequently induces adaptive immune abnormalities, autoantibody production, and persistent antigen presentation [67]. As synovial inflammation develops, large numbers of immune cells infiltrate the joints and mediate inflammatory regulation by fibroblast-like synoviocytes. In particular, macrophages release large amounts of pro-inflammatory factors (such as TNF-α and IL-6) and matrix metalloproteinases, ultimately leading to irreversible bone damage [68].

Regulating abnormal macrophage polarization and inhibiting the inflammatory cascade to block bone destruction are among the research directions for PELN intervention in RA. Existing studies have found that active components (such as gingerols) carried by folic acid-modified ginger-derived exosome-like nanoparticles (FA-GDEVs) can reprogram macrophage polarization in mice [57]. In addition, miRNAs carried by PELNs have the ability to inhibit macrophage inflammatory responses. Studies have shown that pgi-miR6135j contained in ginseng-derived exosome-like nanoparticles (GDNPs) reduces the expression of inflammatory factors in macrophages by inhibiting KRAS activation in synovial macrophages of mice with collagen-induced arthritis (CIA), thereby reducing the phosphorylation levels of downstream c-Jun N-terminal kinase (JNK), extracellular signal-regulated kinase (ERK), and p38 proteins [69].

In addition, excessive osteoclast activation leads to joint erosion in RA. Studies have confirmed that GDNPs inhibit IκBα degradation and JNK phosphorylation; downregulate the transcriptional activities of nuclear factor of activated T-cells, cytoplasmic 1 (NFATc1), and c-Fos/activator protein 1 (AP-1); and reduce the synthesis of functional proteins such as tartrate-resistant acid phosphatase (TRAP) and osteoclast-associated receptor (OSCAR), thereby inhibiting osteoclast differentiation and bone resorption at the source [70] (Figure 2).

### 3.2. Inflammatory Bowel Disease

Inflammatory bowel disease (IBD), which primarily comprises ulcerative colitis (UC) and Crohn’s disease (CD), is an autoimmune intestinal inflammatory disease caused by intestinal barrier disruption and loss of mucosal homeostasis [71]. PELNs can regulate the balance of intestinal immune cells, inhibit inflammatory signaling pathways, and reverse intestinal inflammatory responses [72] (Figure 3).

#### 3.2.1. Improvement of the Immune Microenvironment

IBD is not driven by abnormalities in a single immune cell type but results from the combined effects of persistent innate immune activation, enhanced antigen presentation, and adaptive immune imbalance following intestinal barrier damage [73]. Large numbers of neutrophils, dendritic cells, and macrophages are recruited to inflamed mucosa. While clearing pathogens and damage-associated signals, these cells continuously release inflammatory mediators and further promote the activation of adaptive immune cells, such as T cells, thereby forming an immune cycle in which inflammation continuously amplifies itself [74].

PELNs can limit the excessive effector functions of innate immune cells in IBD, for instance, by reducing the formation of neutrophil extracellular traps (NETs). One mechanism involves miR-5106 in Coptis chinensis-derived exosome-like nanoparticles (Cc-ELNs), which maintains intracellular Zn^2+^ homeostasis by downregulating Slc39a2 protein expression in mouse bone marrow-derived neutrophils [75]. In addition, as a bridge between innate and adaptive immune regulation in IBD, PELNs can inhibit the enhancement of costimulatory signals induced by abnormal dendritic cell activation [76]. A study investigating the effects of broccoli-derived exosome-like nanoparticles (B-ELNs) in a mouse colitis model provides direct evidence: after being internalized by dendritic cells (DCs), B-ELNs not only activate AMP-activated protein kinase (AMPK) and inhibit the mechanistic target of rapamycin complex 1 (mTORC1) signaling pathway, inducing a tolerogenic DC phenotype, but also downregulate the expression of major histocompatibility complex class II (MHC II) and the costimulatory molecules CD80/CD86 [77].

PELNs not only inhibit innate immune activation but may also influence the differentiation fate of T cells. Studies have shown that Portulaca oleracea-derived exosome-like nanoparticles (PO-ELNs) promote the proliferation of Lactobacillus reuteri in the intestines of mice with IBD and increase the levels of indole metabolites. Activation of the aryl hydrocarbon receptor (AhR) in CD4^+^ T cells inhibits Zbtb7b, a master regulator of the CD4^+^ T-cell lineage, and promotes the reprogramming of conventional CD4^+^ T cells into CD4^+^CD8^+^ double-positive T cells. This indicates that regulating adaptive immune cell lineages and reshaping immune responses in colonic inflammatory regions represent another regulatory pathway of PELNs [78].

PELNs can also promote a balance between inflammatory and reparative macrophage phenotypes. GDNPs promote macrophage polarization toward the M2 phenotype by inhibiting AKT/mTOR signaling and enhancing LC3- and Atg7-related autophagic activity. Meanwhile, GDNPs reduce nitric oxide (NO) and NF-κB protein expression in the supernatant of Caco-2 cells, reduce intestinal permeability in mice with IBD, and alleviate mucosal damage. Therefore, autophagy may serve as an important hub linking immunometabolism with PELN-mediated macrophage functional remodeling, thereby promoting tissue repair and the maintenance of homeostasis [58].

#### 3.2.2. Inhibition of Inflammatory Responses

In IBD, PELNs interact with specific receptors on the surfaces of immune cells to promote the secretion of anti-inflammatory cytokines [79]. IL-10, an anti-inflammatory factor secreted by M2 macrophages, can be induced by both grape-derived exosome-like nanoparticles (GELENs) and Atractylodes macrocephala-derived exosome-like nanoparticles (A-ELNs), thereby enhancing the anti-inflammatory capacity of intestinal tissues and protecting the intestinal barrier in mice with IBD [80,81]. In addition, GELNs alleviate colitis by targeting and inhibiting TAB1 via osamiR164d, thereby promoting M2 polarization [82].

The NLRP3 inflammasome can activate caspase-1 and promote the maturation and release of IL-1β and IL-18 [83]. Studies have shown that prune-derived exosome-like nanoparticles (PENVs), after being internalized by macrophages, selectively disrupt the NEK7–NLRP3 interaction, inhibit caspase-1 autocleavage and IL-1β secretion caused by NLRP3 inflammasome formation, and alleviate inflammatory infiltration of the intestinal mucosa in mice with IBD [84].

#### 3.2.3. Intestinal Barrier Repair

The Wnt/β-catenin signaling pathway is an important pathway regulating intestinal stem cell self-renewal, proliferation, and differentiation [85]. GELENs can activate the Tcf4-mediated Wnt/β-catenin pathway, upregulate the target gene Lgr5-EGFP, promote β-catenin accumulation in intestinal epithelial cells and intestinal stem cell differentiation, and repair inflammatory damage, thereby participating in intestinal tissue remodeling in mice with IBD [86].

#### 3.2.4. Regulation of Intestinal Oxidative Stress Responses

Oxidative stress refers to an imbalance between the body’s oxidative and antioxidant systems [87]. Persistent oxidative stress can induce intracellular accumulation of free iron ions and lipid peroxidation. Current studies have demonstrated that Momordica charantia-derived exosome-like nanoparticles (MC-ELNs) can enhance the antioxidant capacity of the intestinal mucosa in mice with IBD, as evidenced by the restoration of redox-related indicators in intestinal tissues following MC-ELN intervention [88].

### 3.3. Type 1 Diabetes Mellitus

Type 1 diabetes mellitus (T1DM) is a chronic disease characterized by persistent impairment of pancreatic β-cell function and severe insulin deficiency caused by autoimmunity and other factors [89]. As β-cell function continues to deteriorate, the body remains in a state of hyperglycemia and metabolic disorder, further inducing oxidative stress, chronic inflammation, and impaired tissue repair [90]. Therefore, the treatment of T1DM requires not only the restoration of pancreatic β-cell function but also amelioration of tissue damage caused by an abnormal metabolic microenvironment.

#### 3.3.1. Improvement of Pancreatic Β-Cell Dysfunction

PELNs can ameliorate pancreatic insufficiency associated with type 1 diabetes. Studies have found that intraperitoneal injection of Trifolium pratense-derived exosome-like nanoparticles (TP-ELNs) can improve serum biochemical indicators and redox status in streptozotocin (STZ)-induced diabetic rats and regulate indicators such as nitric oxide (NO) and total antioxidant capacity (TAC). Meanwhile, in pancreatic tissues, TP-ELNs upregulate the expression of genes associated with β-cell function, pancreatic endocrine cell differentiation and cellular stress regulation, including PDX1, NGN3, and SIRT1 [91]. These results suggest that PELNs can ameliorate pancreatic oxidative stress and regulate the expression of genes associated with β-cell function.

#### 3.3.2. Promotion of Tissue Repair in T1DM

Complications of type 1 diabetes are major determinants of long-term patient prognosis, with chronic wounds and impaired bone tissue repair being typical examples [92]. Persistent hyperglycemia can induce oxidative stress, inflammatory responses, and impair neovascularization, ultimately damaging peripheral tissues [93]. Functional proteins, lipids, and metabolites enriched in PELNs have demonstrated potential application value for promoting chronic wound healing and bone injury repair in diabetic mouse models.

Studies have shown that active components contained in Portulaca oleracea-derived exosome-like nanoparticles (PO-ELNs), including heat shock proteins, linoleic acid, ginkgolic acid, and lipoxins, can eliminate reactive oxygen species (ROS), upregulate ITGB4 and ITGA6 to activate the focal adhesion signaling pathway, and induce PARP1 expression to initiate DNA repair, thereby restoring impaired wound function in diabetic mice [94]. Similarly, mango ginger-derived exosome-like nanoparticles (M-ELNs) accelerate delayed wound healing in diabetic mouse models by promoting keratinocyte migration. In addition to soft tissue wounds, PELNs can ameliorate bone tissue damage caused by diabetes [95]. Viola yedoensis Makino-derived exosome-like nanovesicles (VDNPs) promote M2 macrophage polarization by downregulating NF-κB and achieve a balance between the anti-inflammatory genes (Arg-1 and IL-10) and the pro-inflammatory genes (iNOS and TNF-α), thereby accelerating femoral fracture healing in diabetic rats [96]. These studies suggest that PELNs have the potential to regulate both primary lesions and the comorbid microenvironment in T1DM (Table 3).

Based on Table 3, we conclude that PELN intervention in AIDs exhibits the following characteristics: (1) The administration route can be selected according to the anatomical site of the lesion. For RA and IBD, intervention is mainly performed via intravenous injection and oral gavage, through which PELNs can reach the joints via the systemic circulation or accumulate in the intestine via the digestive tract. For T1DM, intraperitoneal injection is adopted for intervention on the primary injury, whereas local administration is preferable for complications such as skin lesions and fractures [57,69,77]. (2) The intervention efficacy of PELNs is related to the administered dose but is not strictly dose-dependent. Existing preclinical studies indicate that, within a certain dose range, the intervention efficacy of PELNs for AIDs increases with the administered dose. However, some studies have also observed that satisfactory intervention effects can be achieved at low-to-moderate doses, suggesting that the dose–effect relationship is not a simple linear positive correlation. In addition, given the considerable variation in dosage and dosing methods across studies and the lack of a unified standard, the optimal effective dose and safe dose range of PELNs remain to be further defined [69,78,81,88,91]. (3) In preclinical studies of PELN intervention in AIDs, there have been few reports of toxicity to the liver, kidneys, or other vital organs, indicating that PELNs are, to some extent, associated with a favorable safety profile (see the “Safety” column). Moreover, the literature listed in the table does not comprehensively report the compositional makeup of PELNs. Most studies focus on their biological mechanisms of action, evaluating PELNs as a biological or functional unit. This current state of research leaves the pharmacologically active substance basis of PELNs unclear and makes it difficult to meet the requirements for quality control and clinical translation. Therefore, progressively refining the complex components of PELNs will constitute a new direction for their future development.

## 4. Delivery Efficiency of PELNs

Improving the delivery efficiency of PELNs is key to achieving effective intervention in AIDs. PELNs exhibit different potentials in terms of their natural tropism and targeting capabilities.

### 4.1. Effects of Anatomical Structures on the In Vivo Distribution of PELNs

Intravenous injection causes PELNs to pass through the lungs first during blood circulation, followed by accumulation in metabolic organs such as the liver and spleen. Oral administration results in the enrichment of acid-resistant PELNs in the gastrointestinal tract [97]. Intranasal administration preferentially distributes PELNs to the brain and lungs through neural pathways and the respiratory tract. Intramuscular injection results in their distribution to the gastrointestinal tract and liver [98]. This distribution essentially represents passive targeting influenced by different administration routes, the anatomical structures involved, and blood flow pathways, rather than the active targeting capability of PELNs.

### 4.2. Targeting Potential of PELNs

PELNs enriched with different lipid components appear to be selectively internalized by intestinal cells and to exert functional effects. In the intestine, GELNs with a high PA content can be preferentially taken up by Lactobacillus rhamnosus GG (LGG), promoting GELN-carried miRNAs to activate the aryl hydrocarbon receptor (AHR) signaling pathway, thereby inducing intestinal antimicrobial immunity and repair of the intestinal surface barrier [99]. Similarly, grapefruit-derived exosome-like nanoparticles (GF-ELNs) are more likely to migrate from the cecum to the liver [100], which may be associated with the first-pass effect resulting from the metabolism of naringenin in GF-ELNs [101].

Interactions between proteins on the surface of PELNs and membrane proteins on recipient cells also reflect the tropism of PELNs. Mannose-specific binding protein II (lectin) on the surface of garlic-derived exosome-like nanoparticles (GENs) can specifically recognize and bind to the glycosylation modifications of the transmembrane protein CD98, which is highly expressed on the surface of human hepatocellular carcinoma cells (HepG2), thereby mediating efficient internalization of GENs by HepG2 cells [102]. This targeted interaction reveals the tropism of PELNs toward different cells and provides a potential molecular basis for precise immune regulation in AIDs.

### 4.3. Controversies Regarding PELN Delivery and Function

Notably, observations of cellular uptake and intracellular delivery alone are insufficient to establish that PELNs exert functional regulatory effects on recipient cells, and in particular, they cannot determine whether their p-miRNAs silence target genes. Although most studies support the cross-species regulatory function of p-miRNAs, previous studies [69,76] have shown that p-miRNAs in PELNs can act on AIDs mouse models, and p-miRNAs resistant to RNase A have been detected in human plasma [103], the biological mechanisms of p-miRNAs remain controversial: rigorous assays such as RT-qPCR have failed to consistently detect intact p-miRNAs in the plasma and tissues of humans, mice, or bees that have consumed plant-derived foods [104,105,106], and current studies cannot fully exclude differences in detection methods or false-positive interference. Therefore, demonstrating the effects of p-miRNAs requires combining the tissue distribution revealed by in vivo fluorescence tracing with the cross-species regulatory function reflected by RT-qPCR. A study on sea buckthorn-derived exosome-like nanoparticles (SELNs) delivering p-miRNAs across species to promote bone regeneration exemplifies this combined approach: not only was the arrival of SELNs at the femoral epiphysis observed by in vivo imaging, but miRNA sequencing revealed that they are enriched in aau-miR168, and functional experiments confirmed that aau-miR168 promotes osteogenesis in stem cells [107]. Such combined approaches are likely to become the primary means of studying p-miRNA function in the future.

### 4.4. Engineering as a Key to Improving the Intervention Efficiency of PELNs

To further improve the intervention precision of natural PELNs, researchers are currently considering modifications in two aspects: encapsulating exogenous active molecules within PELNs and introducing surface modifications to enhance targeting (Figure 4).

Exogenous loading into PELNs can be divided into passive diffusion (co-incubation) and active loading (electroporation, ultrasound, freeze–thaw, surfactant permeabilization, and extrusion) [108,109]. Passive diffusion relies on concentration gradients to drive the spontaneous penetration of drugs into the lipid bilayer [110]. For example, vancomycin at a high concentration spontaneously migrates into and integrates with garlic-derived exosome-like nanoparticles (GENs) [111]. This method avoids membrane damage caused by physical techniques [112]. In addition, nonpolar regions in the structures of drug molecules, such as curcumin, paclitaxel, and heparin, can be anchored or embedded in the PELNs lipid bilayer through their inherent hydrophobicity. For example, GELNs can achieve stable loading of siRNA-CD98 through co-incubation and exhibit siRNA delivery efficiency comparable to that of commercial liposomal transfection reagents in colon cancer CT-26 cells and RAW264 macrophages [113].

Active drug loading uses physical perturbation to transiently open membrane structures and improve loading efficiency. These approaches include the formation of transient hydrophilic pores (electroporation), the introduction of shock waves, liquid jets (ultrasound), aqueous phase transitions (freeze–thaw), the addition of low concentrations of surfactant molecules (surfactant permeabilization), and mechanical compression to induce membrane lipid reorganization (extrusion) [114]. For example, the loading efficiency of doxorubicin (Dox) in GELNs treated with ultrasound combined with co-incubation can reach 95.9% ± 0.26%, while the thin-film hydration–ultrasound extrusion method can also achieve a doxorubicin encapsulation efficiency of 75% [115].

Surface modification can introduce tissue-specific ligands through chemical conjugation, electrostatic adsorption, and membrane hybridization to improve targeting and barrier penetration [116]. One study used an amidation reaction to conjugate heparin-cRGD to the amino groups of lemon-derived exosome-like nanoparticles (LELNs), thereby enhancing ovarian cancer penetration and antitumor effects [114]. Electrostatic adsorption is also a commonly used approach, in which the membrane potential of PELNs is adjusted to enable binding to target mRNA carrying an opposite charge. For example, pomatto et al. modified sweet orange-derived exosome-like nanoparticles (CS-ELNs) using polycations to achieve loading of SARS-CoV-2 RBD mRNA [117]. In addition, in vaccine development, HELNs derived from neem and other sources have been combined with chitosan and polyethylene glycol-modified graphene oxide to construct composite carriers, effectively delivering siRNA from engineered vesicles into MCF7 cells and blocking the biological function of target genes [118]. Furthermore, PELNs can be fused with chemical liposome membranes to enhance their functions. For example, hydrangea leaf-derived exosome-like nanoparticles (HML-ELNs) were fused with specific terpene-based liposomal membranes to ameliorate UV-induced skin damage [119] (Table 4).

With the advancement of PELN research, the application value of PELN engineering in AIDs intervention has become increasingly prominent. In a study of rheumatoid arthritis, FA-PEG2000-Chol was modified onto the surface of GELNs [57]. Through folate ligand-mediated recognition of folate receptors on activated M1 macrophages, this modification enabled GELNs to actively target sites of joint inflammation. Grapefruit exosome-like vesicles loaded with the anticancer drug CX5461 (GEV@CX5461) were combined with gingival mesenchymal stem cell membranes expressing the CCR6 receptor on their surfaces to synthesize the FV@CX5461 complex. This complex not only maintained the stability of CX5461 but also exploited the specific recognition between the CCR6 receptor on the stem cell membrane surface and CCL20, which is highly expressed at inflammatory sites, to precisely guide the vesicles to inflammatory skin lesions, providing a new approach for targeted intervention in autoimmune skin diseases [124].

In addition, engineering modifications have significantly improved the colon-targeting capability of PELNs. For example, red cabbage-derived exosome-like nanoparticles (RELNs), following surface conjugation with hyaluronic acid, can simultaneously enhance their targeting of intestinal epithelial cells and immune cells [125]. Another study developed a more comprehensive delivery system: a biomimetic nanocomposite consisting of large mesoporous silica nanoparticles and GELNs. In this composite, ultrasound serves as a physical energy input to generate cavitation effects and mechanical forces, which deform and open the soft GELN membranes, enabling them to re-encapsulate the rigid silica nanoparticles and thereby form a tightly bound core–shell structure through noncovalent interactions [126].

## 5. Standardized Preparation of PELNs

Standardization is an essential step toward the application of PELNs for AIDs intervention and clinical translation. The biological properties of PELNs are susceptible to their sources and preparation procedures. In the transition from basic research to large-scale clinical production, it is necessary to determine whether PELNs obtained from different plant batches can maintain consistent cargo composition and purity while retaining favorable safety profiles (Figure 5).

### 5.1. PELN Pretreatment and Isolation

Differences in the initial plant materials can lead to biological variations in PELNs. The current challenge is that, even within the same plant species, various variables associated with PELNs derived from different growing environments and tissue parts are difficult to control precisely. For example, the concentration of Hibiscus syriacus leaf-derived exosome-like nanoparticles (HSL-ELNs) is twice that of flower-derived PELNs, and their metabolomic profiles also differ significantly. Similar differences have been observed in the protein content of potato root-derived exosome-like nanoparticles (PR-ELNs) and potato peel-derived exosome-like nanoparticles (PP-ELNs), with root-derived EVs accounting for 42% and peel-derived EVs for 25%. Another experiment found that geographical environments can lead to differences in the lipid composition of GELNs, resulting in differential uptake responses by B cells. Therefore, even when the plant species are identical, the variability in outcomes caused by differences in PELN raw materials must be strictly defined. Isolation methods also affect PELN quality. In a study of Morinda officinalis root-derived exosome-like nanoparticles (MO-ELNs), the conventional method yielded approximately 1.41 × 10^9^ particles/g, whereas the enzymatic digestion method yielded approximately 4.35 × 10^9^ particles/g. The particle size distributions and cargo compositions obtained using the two methods differed, and metabolomic analysis alone identified 182 differential metabolites. Therefore, standardization of PELN raw materials requires comprehensive control over plant species, tissue parts, and preparation methods, as any step may affect the functional properties of PELNs [127].

Temperature and pH also affect PELN extraction efficiency. A temperature of 4 °C helps maintain the integrity of PELN membranes but reduces the activity of cell wall-degrading enzymes, which is unfavorable for PELN release [128]. Although high temperatures can improve enzymatic digestion efficiency, they may disrupt protein structures and alter cargo composition. Changes in the pH of the PELN mixture can affect vesicle size. In addition, pectinase-based PELN pretreatment requires multiple rounds of condition optimization. For example, cold soaking results in low release efficiency and requires more enzymes and time [129,130]. In contrast, high-temperature treatment with added enzymes requires a shorter treatment duration, thereby protecting PELN proteins and membrane structures [128].

### 5.2. Evaluation of PELN Purity

Although the MISEV2023 report primarily focuses on M-Exos, its guidelines are also applicable to the standardized evaluation of PELNs’ purity [131]. Current mainstream methods for PELN isolation include differential ultracentrifugation, density gradient centrifugation, ultrafiltration, size-exclusion chromatography (SEC), polymer precipitation, and immunoaffinity capture [132,133]. It is worth noting that, in centrifugation- and precipitation-based methods, the supernatant obtained after mechanical disruption of plant tissues contains large amounts of lipoprotein complexes, protein aggregates, and potential contaminants [134]. These non-PELN substances are difficult to exclude during nanoparticle tracking analysis (NTA), leading to overestimation of particle purity. Sucrose or iodixanol can be used to further separate impurities, but the efficiency remains unknown [131].

Current PELNs purity evaluations mainly employ multiparametric analyses. The protein-to-particle ratio is used as an indicator for assessing protein impurities, protein aggregation, or nonspecific co-precipitation. In addition, detection of the PELN marker proteins TET8/TET9 serves as a reference for evaluating PELN cargo, while PELN characteristics can also be identified by microscopy [135].

For characterization and identification, different detection techniques may yield different results. Transmission electron microscopy and scanning electron microscopy are commonly used methods. Electron beams penetrate PELNs to provide rapid and intuitive information, revealing a “cup-shaped” or “concave” appearance under the microscope [136]. Recent studies have shown that these shapes are associated with vesicle collapse caused by drying during negative staining sample preparation, potentially leading to discrepancies between the microscopic morphology of PELNs and their actual state. In contrast, cryogenic electron microscopy (cryo-EM) preserves PELNs in vitreous ice [137], while atomic force microscopy (AFM) preserves them in a liquid phase. Neither method requires dehydration or fixation, allowing PELNs to exhibit spherical morphologies close to their native state. AFM can also provide mechanical data, including three-dimensional morphology, elasticity, and adhesion, thereby improving the accuracy of individual PELN assessment. Recently, Chukhchin et al. used AFM to observe spherical PELN structures with diameters of 655 nm under near-native conditions [138].

### 5.3. Safety of PELNs

Theoretically, owing to their natural origins, PELNs are more consistent with the requirements of an ideal drug delivery system characterized by low toxicity and non-immunogenicity than nanoparticles derived from animals, bacteria, or synthetic materials [80,139]. Multiple safety assessments of PELNs for AIDs intervention discussed above, including serological and pathological indicators, have shown that PELNs exhibit low toxicity both in vivo and in vitro (Table 3). Currently, M-Exos, such as mesenchymal stem cell-derived extracellular vesicles (MSCs-EVs), have an established safety assessment system covering DNA genotoxicity, human hemolysis, infection, innate immune system defense, and endotoxin-related effects, as well as clinical symptoms, hematology, clinical biochemistry, and histopathology [140]. This existing standardized safety review system provides an important reference for PELN safety assessment. Although no toxicity reports from human clinical trials of PELNs are currently available, existing animal experiments can help clarify future evaluation directions.

PELNs still lack sufficient data to support the establishment of evaluation criteria for the immunogenicity of existing intervention regimens. The evaluation approaches for short-term and long-term administration differ. Short-term data exhibit a “route-dependent” characteristic, meaning that the body’s response primarily depends on the intervention strategy [141]. Oral PELNs are rapidly cleared in vivo, undergo digestion by gastric fluids, and exhibit low short-term immunogenicity, making them a preferred option for maintaining microbiota stability [142]. Tail vein injection offers slightly lower safety and suitability than oral administration. For example, following tail vein injection of edible tea flower-derived exosome-like nanovesicles, surface proteins and polysaccharides can nonspecifically bind to complement components and activate the C3 complement system, resulting in mild hepatotoxicity [143]. DSPE-PEG2000 surface modification is currently being used to address C3 complement system activation [144].

Data on the toxicity of long-term repeated administration remain an industry-wide gap. Repeated interventions lasting several weeks to several months require greater attention to the recipient species and the degree to which the administration duration matches the clinical treatment cycle. Immunogenicity is the primary concern for long-term administration. Anti-drug antibodies may alter drug efficacy or trigger immune responses and therefore require continuous monitoring throughout the entire development cycle. Because the relevant species and immunogenicity characteristics have not yet been established, long-term toxicological data for PELNs remain unavailable. Therefore, toxicological studies of long-term PELN intervention are crucial for their industrial translation [145].

Endotoxin and microbial contamination are among the safety risks that require attention in the cross-kingdom application of PELNs. Unlike M-Exos, PELNs are mainly derived from edible plants, resulting in a relatively lower risk of introducing zoonotic or human pathogens [116]. In contrast, RNA-mediated off-target effects may be a more important concern in PELN-mediated cross-kingdom regulation. Such off-target effects may induce immunogenicity and abnormally activate innate immune responses, and this issue may also arise during PELN delivery [146]. Current optimization strategies focus on two aspects: (1) identifying potential off-target miRNAs in preliminary experiments—for example, immunostimulatory motifs such as 5′-UGU-3′ and 5′-UGUGU-3′ can be eliminated, or AGO2-RIP immunoprecipitation can be used to clarify the specificity of p-miRNA binding to mammalian mRNAs [147]—and (2) reducing nonspecific targeting through appropriate RNA chemical modifications—mechanistic studies have demonstrated that 2′-O-methylation of the siRNA seed region can create steric hindrance within the AGO protein complex; this hindrance reduces weak base-pairing interactions between the seed region and non-target mRNAs, thereby improving the precision of gene therapy [148]. However, in PELNs, 2′-O-methylation of p-miRNAs occurs mainly at the 3′-terminal nucleotide rather than in the seed sequence region. Therefore, whether PELNs can produce off-target suppression effects similar to those resulting from 2′-O-methylation of the siRNA seed region requires further validation [149].

### 5.4. PELN Storage Methods

Temperature is a key factor affecting PELNs’ membrane structure and the stability of biomolecules. Taking GELENs as an example, storage at −80 °C can maintain protein content and particle numbers at levels close to those of fresh GELENs over a three-month storage period. Storage at −20 °C can better preserve GELEN particle concentration and protein stability in the short term but is unsuitable for storage exceeding two months. At higher storage temperatures (4 °C and 25 °C), significant membrane lipid degradation and sphingolipid metabolic imbalances occur, indicating that room-temperature conditions cannot adequately inhibit oxidation, hydrolysis, and enzymatic reactions in PELNs [150].

Storage at −80 °C relies heavily on an ultralow-temperature cold chain and can ensure the stability of raw materials. However, clinical end use requires more practical formulated products. The addition of cryoprotectants/preservatives (trehalose and LEVs-TMO) can extend the preservation of PELN physical structure and function against temperature-induced disturbances [151]. Therefore, for PELNs requiring long-term low-temperature storage, repeated freeze–thaw cycles should be minimized, and appropriate protectants should be considered to maintain membrane structure. For PELNs requiring further extension of storage time or improved transportation convenience, lyophilization also has potential applications, such as lyophilized sprays and freeze-dried powders. Studies have shown that two months represents the optimal storage duration for lyophilized GDNPs at room temperature, whereas total protein and RNA contents begin to decline after three months [151]. In addition, to accommodate different needs, Cai et al. loaded Drynaria roosii-derived exosome-like nanoparticles (DR-ELNs) into GelMA microspheres and embedded them in a sinomenine-containing HA/HPMC thermosensitive hydrogel. This sustained-release method can prolong the intervention period for synovial inflammation and cartilage damage in rat models of RA [152].

## 6. Future Trends in PELNs Formulation Development

As plant-specific raw materials for drug preparation, PELNs have demonstrated significant potential in targeted delivery and remodeling of the immune microenvironment. They have a “dual role” in immune regulation within the body and offer considerable promise for the development of clinical formulations [153].

Standardization and GMP-compliant production are key steps in the industrial development of PELNs. Although organizations such as the International Society for Extracellular Vesicles (ISEV) and the European Network on Microvesicles and Exosomes in Health and Disease (ME-HaD) have established relevant regulations to promote the clinical translation of EVs [154,155], GMP regulatory rules for PELNs have not yet been established. Standardization therefore remains a major obstacle to their clinical translation [156].

Against this background, researchers are advancing standardization efforts from two aspects: raw material control and production processes. In terms of raw materials, artificial cultivation using a TIBS culture system is expected to reduce batch-to-batch quality instability in plant materials used for PELN production [157]. In terms of production processes, multiple isolation techniques have been combined [158], and key parameters such as enzyme concentration, pH [159], and temperature have been controlled in an effort to minimize residual non-vesicular components and impurity interference. In addition, given the complex composition of PELNs, specific guidelines for their safety and potency, as well as dedicated GMP requirements, need to be established independently [160]. In the future, the PELNs preparation system should be optimized in several aspects: emphasis should be placed on establishing a standardized PELN evaluation system, for example, by using principal component analysis (PCA) to assess differences among samples [161]. Refined and scalable biomanufacturing processes should be strengthened, such as achieving more precise isolation through light, magnetic, electrical, and acoustic approaches, as well as novel methods such as oscillating microbubble arrays [162], and PELNs should be combined with gene editing to provide a feasible pathway for intervention and the development of targeted strategies against AIDs [163].

## 7. Conclusions

As natural nanodelivery carriers unique to plants, PELNs achieve cross-kingdom delivery and precise regulation of their bioactive cargo through their distinctive nanovesicular structures, demonstrating considerable potential in suppressing inflammatory responses and remodeling the immune microenvironment. Although the clinical translation of PELNs still faces certain challenges, continued advances in delivery efficiency, standardization, and production processes are expected to establish PELNs as a new strategy for targeted and precise intervention in AIDs.

## Figures and Tables

**Figure 1:**
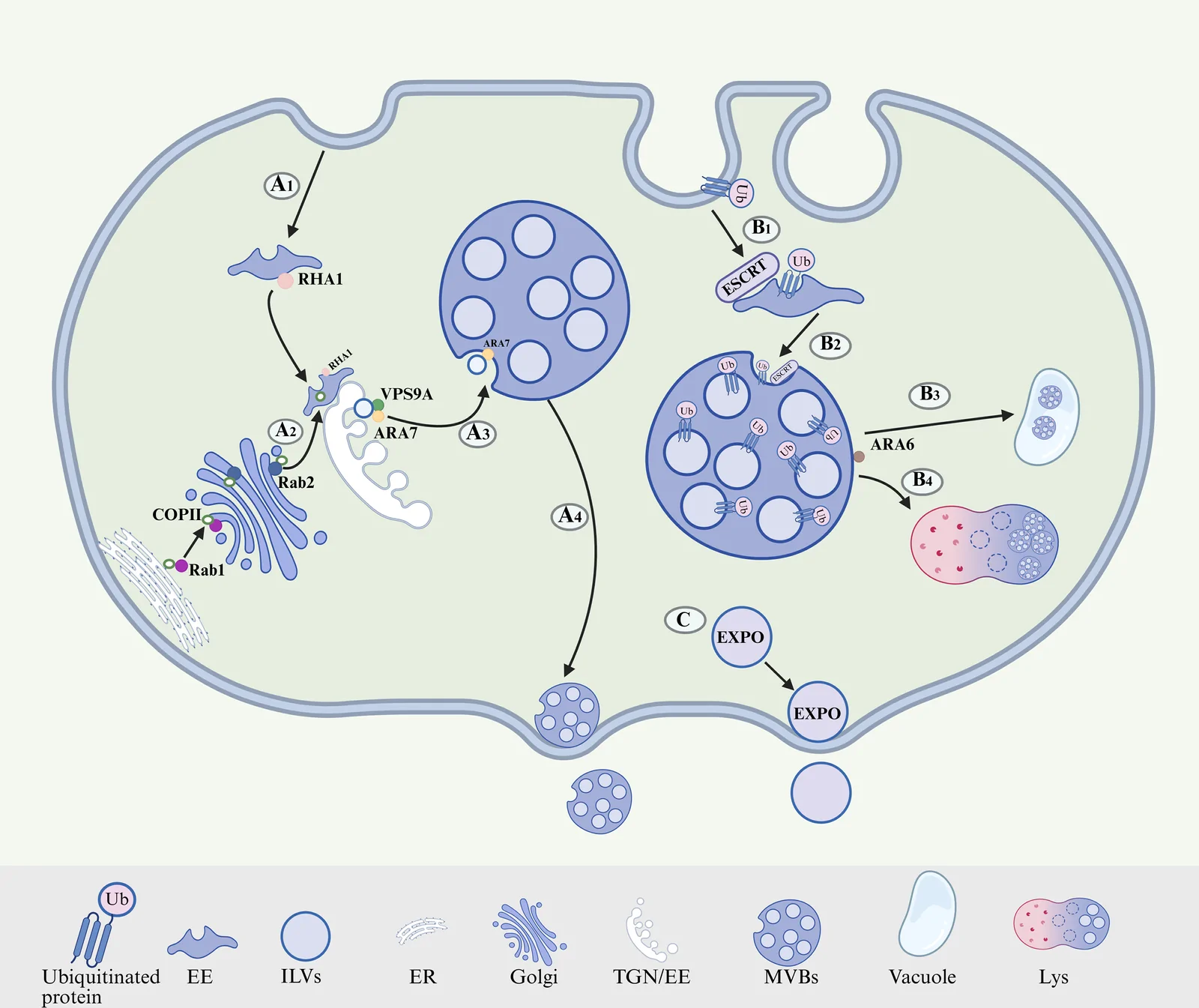
The biogenesis pathway of PELNs. (**A_1_**–**A_4_**): This process involves Rab1/Rab2-dependent vesicle trafficking from the Golgi apparatus to endosomes, followed by RHA1, VPS9A and ARA7-mediated maturation of MVBs and subsequent extracellular vesicle release. (**B_1_**–**B_4_**): This process illustrates ESCRT complex-mediated intraluminal vesicle and multivesicular body formation via ubiquitinated receptor recognition, as well as ARA6-dependent targeting of multivesicular bodies to the vacuole/lysosome for degradation. (**C**). This process illustrates EXPO organelles fusing with the plasma membrane to release PELNs.

**Figure 2:**
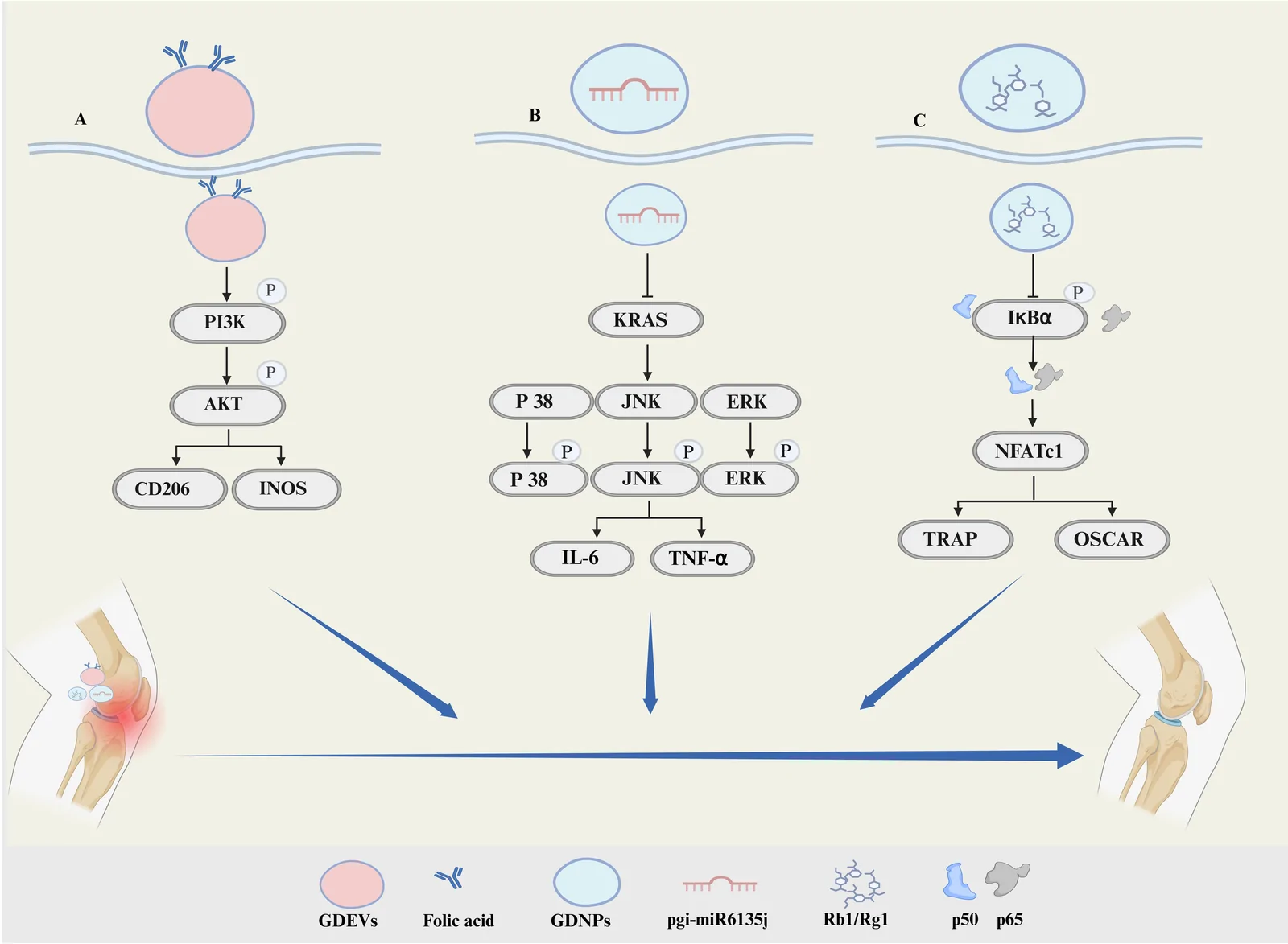
Therapeutic mechanisms of PELNs in rheumatoid arthritis. (**A**). Folic acid-modified GDEVs activate the PI3K/AKT pathway, regulating the polarization of macrophages from the M1 to the M2 phenotype (CD206/iNOS). (**B**). pgi-miR6135j in GDNPs inhibits KRAS expression and the activation of the p38/JNK/ERK pathway, reducing the expression of inflammatory cytokines IL-6 and TNF-α. (**C**). Rb1/Rg1 in GDNPs suppresses IκBα expression, thereby inhibiting NFATc1 transcription and downregulating the translation of osteoclast markers TRAP and OSCAR.

**Figure 3:**
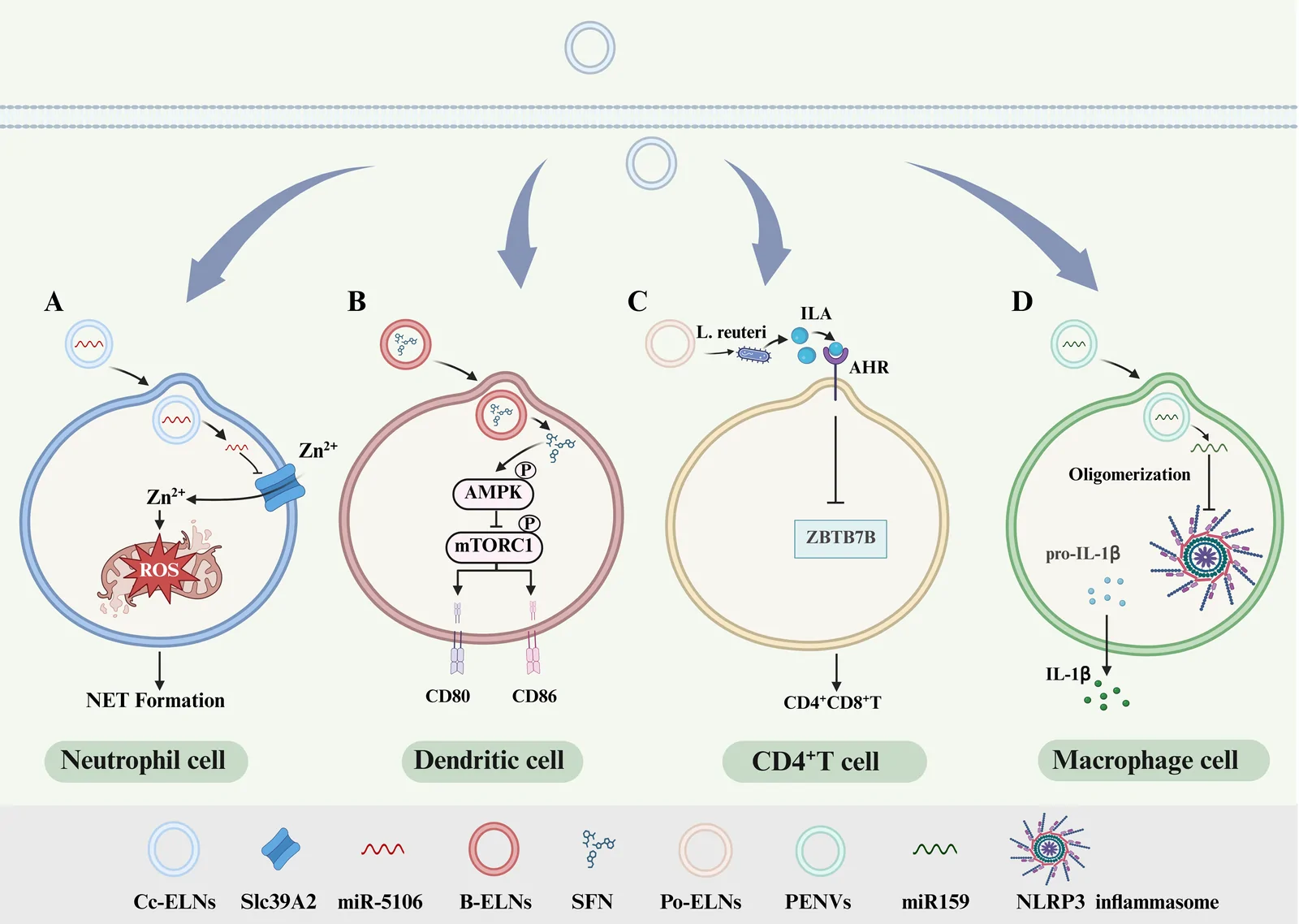
Therapeutic mechanisms of PELNs in IBD. (**A**). miR-5106 contained in Cc-ELNs inhibits the expression of Slc39A2, reduces Zn^2+^ influx, and thereby decreases mitochondrial ROS production and NET formation. (**B**). SFN in B-ELNs suppresses mTORC1 signaling and upregulates the expression of costimulatory molecules CD80/CD86 on the surface of dendritic cells. (**C**). Po-ELNs elevate the level of ILA to activate AHR, inhibit ZBTB7B expression in CD4^+^ T cells, and induce the reprogramming of CD4^+^ T cells into the CD4^+^CD8^+^ double-positive phenotype. (**D**). miR159 derived from PENVs inhibits NLRP3 inflammasome assembly and IL-1β maturation in macrophages.

**Figure 4:**
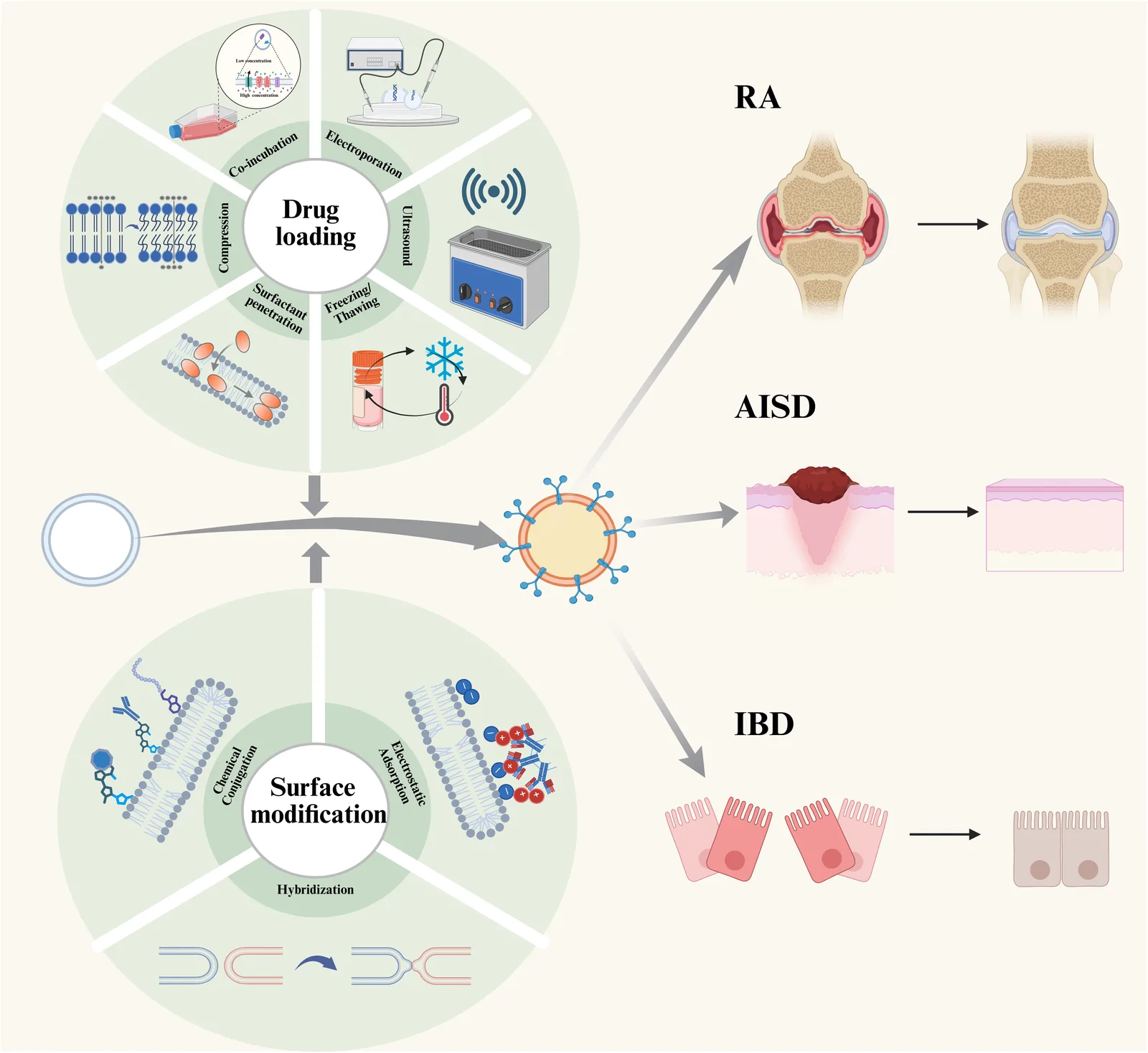
Engineering strategies of PELNs for enhanced therapeutic efficacy in AIDs. This figure shows that PELNs are engineered through drug loading (co-incubation, electroporation, etc.) and surface modification (conjugation, adsorption, hybridization), targeting inflamed sites in RA, AISD, and IBD to exert anti-inflammatory and reparative effects.

**Figure 5:**
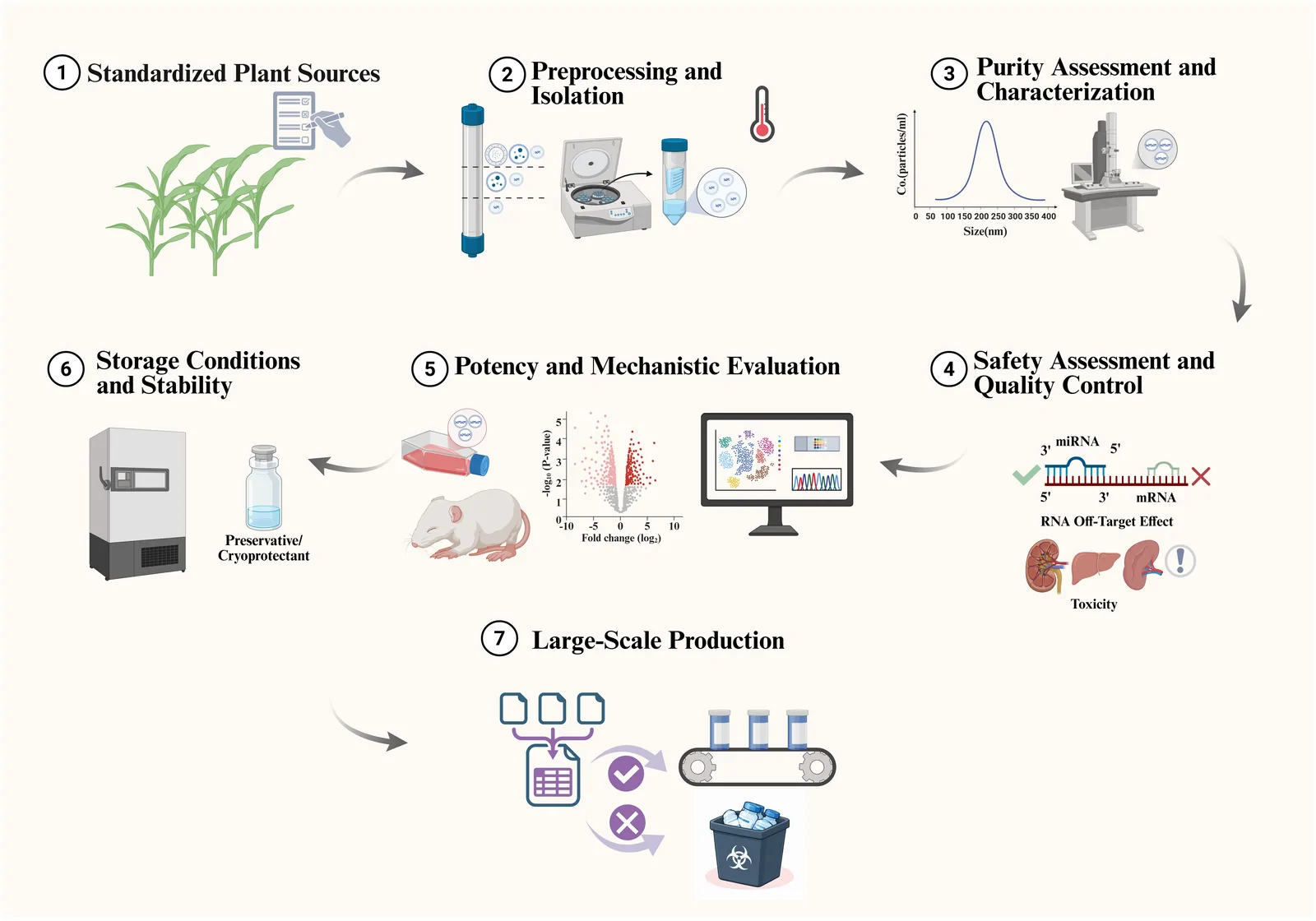
Schematic illustration of the standardization and large-scale production workflow for PELNs. The workflow comprises seven key steps: (**1**) Standardized plant sources. (**2**) Preprocessing and isolation. (**3**) Purity assessment and characterization. (**4**) Safety assessment and quality control. (**5**) Potency and mechanistic evaluation. (**6**) Storage conditions and stability. (**7**) Large-scale production.

**Table 1:** Terminology and definitions of plant-derived vesicles.

Term	Full Name	Scope of Application
EVs	Extracellular Vesicles	General term for extracellular vesicles
PDVs	Plant-Derived Vesicles	General term for plant-derived nanovesicles
PEVs/PDEVs	Plant Extracellular Vesicles/Plant-Derived Extracellular Vesicles	Naturally secreted vesicles directly isolated from plant apoplastic fluids
PDNVs	Plant-Derived Nanovesicles	Vesicles obtained through processing of plant tissues, such as grinding or juicing
PDVLNs	Plant-Derived Vesicle-Like Nanoparticles	
PELNs	Plant-Derived Exosome-Like Nanovesicles	

**Table 2:** Compositional comparison between PELNs and M-Exos.

Category	Aspect of Comparison	PELNs	M-Exos	Ref.
Lipids	Composition	PC, PE, PI, PS, PA	Phospholipids Saturated phosphatidylcholine	[28]
	Structural	Higher membrane stability	Higher membrane fluidity	[29,30,31]
	Function	PA can mediate immune modulation	/	[32,33,34]
Proteins	Marker protein	PEN1, PEN3, TET-8, TET-9	TSG101, CD9, CD63, CD81	[41,45,46]
	Function	Structural framework, targeting, plant immunity, lipid sorting	Same as PELNs Intercellular signal transduction	[37,38,39,40]
Non-coding RNAs	microRNA	Nuclear maturation 3′-end methylation High stability	Stepwise nuclear Cytoplasmic processing Lack 3′ methylation Lower stability	[50,51,52,53,54,55]
	siRNA	Naturally generated Cross-kingdom potential	Artificial synthesis For experimental or therapeutic	[38]
	Mechanism of Action	Competitive inhibition Allosteric regulation Protein interaction interference	/	[60,61,62,63,64]

**Table 3:** Intervention Applications and Mechanisms of PELNs in AIDs.

Disease	Disease Model	Source	Isolation Characterization Methods	Administration Route and Dosage	Mechanism	Result	Safety	Ref.
RA	BMDMs CIA mice	Ginger	Isolation: DC+UC+SDG Characterization: 1. Vesicle morphology: TEM 2. Particle size measurement: NTA (GDEVs: 241 nm. FA-GDEVs: 268 nm) Zeta potential: GDEVs: −26.57 mV. FA-GDEVs: −22.63 mV	In vitro: Administration route: co-incubation (co-incub) Dose: 150 µg/mL Frequency: 48 h In vivo: Administration route: tail vein injection (i.v.) Dose: FA-GDEV/GDEV (1.5 mg/kg) Frequency: Once every 3 days, for a total of 7 injections	FA-GELNs drive macrophage polarization from M1 to M2	Targeting inflammatory macrophages	1. No cytotoxicity 2. No hemolysis 3. No pathological damage 4. Normal body weight 5. Normal spleen/thymus index	[57]
	BMDMs CIA mice	Ginseng	Isolation: DC+UC+SDG Characterization: 1. Vesicle morphology: TEM 2. Particle size measurement: DLS (0.124–0.132)	In vivo: Administration route: oral gavage (i.g.) Dose: 5 mg/kg and 10 mg/kg High doses achieve better intervention efficacy Frequency: Once every day, 18 days	GDNPs-miR6135j targets KRAS to suppress MAPK signaling	Inhibiting synovial macrophage inflammation	1. No liver/kidney function impairment (ALT, AST, Cr, BUN) 2. No pathological damage	[69]
	BMDMs	Ginseng	Isolation: DC+UC+SDG Characterization: 1. Vesicle morphology: TEM 2. Particle size measurement: NTA (4.18 × 10^11^ particles/0.1 g) 3. Zeta potential (−18.8 mV)	In vitro: Administration route: co-incub Dose: 1 µg/mL Frequency: 4–7 days	GDNPs block NF-κB and MAPK/AP-1 pathways	Inhibiting osteoclast-mediated bone erosion	/	[70]
IBD	Neutrophils DSS-induced colitis mice	Coptis chinensis	Isolation: DC+UC+SDG Characterization: 1. Vesicle morphology: TEM 2. Particle size measurement: NanoFCM	In vitro: Administration route: co-incub Dose: 20 µg/mL Frequency: 24 h In vivo: Administration route: intraperitoneal injection (i.p.) Dose: 50 µg/mice/day Frequency: 8 days	Cc-ELNs-miR-5106 downregulates Slc39a2 to suppress NET formation	Modulating neutrophil function	1. Normal body weight 2. Normal organ indices 3. No liver/kidney function impairment (ALT, AST, TP, CREA, UREA) 4. No pathological damage	[76]
	BMDCs, DCs DSS-induced colitis mic	Broccoli	Isolation: DC+UC+SDG Characterization: 1. Vesicle morphology: TEM 2. Particle size measurement: DLS (32.4 nm) 3. Zeta potential: −17.1 mV	In vitro: Administration route: co-incub Dose: / Frequency: 24 h In vivo: Administration route: i.g. Dose: 250 µg/mice/day Frequency: 10 days	B-ELNs activate AMPKto inhibit mTORC1	Regulating dendritic cell activation	/	[77]
	DSS colitis model	Portulaca oleracea	Isolation: DC+UC+SDG Characterization: 1. Vesicle morphology: Cryo-EM, TEM 2. Particle size measurement: DLS (160 nm) 3. Zeta potential: −31.4 mV	In vivo: Administration route: i.g. Dose: 50 or 100 mg/g High doses achieve better intervention efficacy Frequency: Once daily for 5 days	PO-ELNs reprogram CD4^+^T cells into CD4^+^CD8^+^ double-positive T cells	Increase the expression of the anti-inflammatory cytokine.	1. No pathological damage 2. Normal liver/kidney function (ALT, AST, CREA, Urea)	[78]
	RAW264.7 Caco-2 cell DSS-induced colitis mice	Ginseng	Isolation: DC+UC+SDG Characterization: 1. Morphology: TEM 2. Particle size measurement: Zetasizer (253.61 nm) 3. Zeta potential: /	In vitro: Administration route: co-incub Dose: 5–20 μg/mL Frequency: 24 h In vivo: Administration route: i.g. Dose: 5 mg/mL or 10 mg/mL Frequency: Once daily for 10 days	GDNPs inhibit AKT/mTOR to induce autophagy	Inducing macrophage autophagy	No cytotoxicity	[58]
	RAW264.7 macrophages DSS-induced colitis mice	Grapefruit	Isolation: DC+UC+SDG Characterization: 1. Morphology: TEM 2. Particle size measurement: DLS (210.8 ± 48.62 nm) 3. Zeta potential: −49.2 to −1.52 mV	In vitro: Administration route: co-incub Dose: 2–60 µg/mL Frequency: 24 h In vivo: Administration route: i.g. Dose: 10 mg/kg/d Frequency: Once daily for 7 days	GF-ELNs upregulates macrophage anti-inflammatory factors	Enhancing anti-inflammatory capacity	1. No apoptosis 2. Normal liver/kidney function indices (ALT/AST/IFN-γ) 3. No changes in intestinal morphology	[80]
	BMDMs PMA-THP-1 cells DSS-induced colitis mice	Atractylodes macrocephala	Isolation: DC+UC+SDG Characterization: 1. Morphology: TEM 2. Particle size measurement: NanoFCM (90.61 ± 19.66 nm)	In vitro: Administration route: co-incub Dose: 0.1–0.5 μg/mL, 24 h In vivo: Administration route: i.g. Dose: 0.5–2 mg/kg/d High doses achieve better intervention efficacy Frequency: Once daily for 5 days	A-ELNs modulate Th17 cell response and up-regulate IL-10	Restore gut microbiota balance	1. No cytotoxicity 2. No hemolysis 3. Normal body weight 4. No pathological damage 5. No apoptosis 6. Normal liver/kidney function (ALT, AST, TP, CREA, UREA)	[81]
	RAW264.7 macrophages DSS colitis model	Ginger	Isolation: DC+Exosome isolation kit Characterization: 1. Vesicle morphology: TEM 2. Particle size measurement: NTA (163 ± 28 nm) (2.1 × 10^10^ particles/mL) 3. Zeta potential: −23.7 mV	In vitro: Administration route: co-incub Dose: 0–80 µg/mL Frequency: 24 h In vivo: Administration route: i.g. Dose: 9µg/mice/day Frequency: 8 days	GELNs deliver osa-miR164d to macrophages and downregulate NF-κB.	Promoting anti-inflammatory macrophages	No cytotoxicity	[82]
	BMDMs PMA-THP-1 cells DSS-induced colitis mice	Prune	Isolation: DC+UC+SDG Characterization: 1. Morphology: TEM 2. Particle size measurement: NTA (124 nm) 3. Zeta potential: −27 mV	In vitro: Administration route: co-incub Dose: BMDMs (1 × 10^7^/mL) and PMA-THP-1 cells (3 × 10^7^/mL) In vivo: Administration route: i.g. Dose: DSS model (0.5 × 10^10^) TNBS model (1.5 × 10^10^/mL) Frequency: DSS model (10 days); TNBS model (7 days)	PENVs disrupt NEK7-NLRP3 interaction	Inhibiting NLRP3 inflammasome	1. Normal body weight 2. Normal colon length 3. Normal liver/kidney function (ALT, AST, CREA, Urea) 4. Normal organ indices 5. No pathological damage	[84]
	CT26 cell Lgr5^+^ stem cells DSS-induced colitis mice	Grape	Isolation: DC+UC+SDG Characterization: 1. Morphology: TEM 2. Particle size measurement: DLS (380.5 ± 37.47 nm) 3. Zeta potential: −26.3 ± 8.14 mV	In vitro: Administration route: co-incub Dose: 20 or 40 μg/mL In vivo: Administration route: i.g. Dose: 2 mg/mice/day Frequency: 2 days	GELENs activates Wnt/β-catenin signaling	Repairing intestinal barrier	/	[86]
	BMDCs DCs DSS-induced colitis mice	Momordica charantia	Isolation: DC+UC+SDG Characterization: 1. Vesicle morphology: TEM 2. Particle size measurement: DLS (132.03 ± 9.2 nm) 3. Zeta potential: −11.19 ± 3.07 mV 4. PDI: 0.343 ± 0.039	In vitro: Administration route: co-incub Dose: 50–800 µg/mL Frequency: 24 h In vivo: Administration route: i.g. Dose: 10–40 mg/kg High doses achieve better intervention efficacy Frequency: 5 days	MC-ELNs enhance cellular antioxidant capacity	Regulating oxidative stress	1. No cytotoxicity 2. No apoptosis	[88]
Type 1 Diabetes	STZ-induced T1D model	Trifolium pratense	Isolation: DC+PEG Characterization: 1. Morphology: SEM 2. Particle size measurement: DLS (167.3 nm) 3. Zeta potential: −23.9 mV 4. PDI: 0.33	In vivo: Administration route: i.p. Dose: 100–400 μg/kg Optimal dose for blood glucose reduction: 400 μg/kg Optimal dose for serum insulin and C-peptide: 100 μg/kg Optimal dose for pancreatic gene expression: 200 μg/kg Frequency: Once every day, 28 days	TP-ELNs regulate pancreatic-related genes	Protecting pancreatic function	1. No cytotoxicity 2. No pathological damage	[91]
	HaCaT HSF THP-1 cell T1DM wound model	Portulaca oleracea	Isolation: DC+UC+SDG or PEG Characterization: 1. Morphology: TEM 2. Particle size measurement: NTA (100–200 nm) 3. Zeta potential: −30.7 to −37.9 mV	In vitro: Administration route: co-incub Dose: 5–40 μg/mL Frequency: 24–72 h In vivo: Administration route: topical application of hydrogel (topical) Dose: / Frequency: once	Po-DENs clear ROS and activate DNA repair	Promoting wound healing	1. No cytotoxicity 2. No hemolysis 3. No pathological damage	[94]
	STZ-induced Diabetic wound model	Mango ginger	Isolation: DC+PEG Characterization: 1. Morphology: SEM 2. Particle size measurement: DLS (324.7 ± 28.3 nm) 3. Zeta potential: −16 to −33 mV	In vitro: Administration route: co-incub Dose: 30–60 μg/mL Frequency: 8–24 h In vivo: Administration route: topical Dose: / Frequency: once	ELNs downregulate KSRP and stabilize FSTL1 mRNA	Promotes keratinocyte migration and wound healing	1. No cytotoxicity 2. No pathological damage	[95]
	BMDMs BMSCs Diabetic femoral fracture SD rats	Viola Yedoensis Makino	Isolation: DC+UF+SEC Characterization: 1. Morphology: TEM 2. Particle size measurement: NTA (128.1 nm) (2.135 × 10^10^)	In vitro: Administration route: co-incubation Dose: 200 µg/mL Frequency: 24 h In vivo: Administration route: i.g. Dose: 15 μL DPC-3/VDNPs hydrogel Frequency: once	VDNPs downregulation of NF-κB promotes M2 macrophage polarization	Promotes diabetic fracture healing	1. No cytotoxicity 2. No hemolysis 3. No pathological damage	[96]

Note: DC, differential centrifugation; UC, ultracentrifugation; SDG, sucrose density ultracentrifugation; TEM, transmission electron microscopy; NTA, nanoparticle tracking analysis; UF, ultrafiltration; SEC, size-exclusion chromatography; PEG, precipitation.

**Table 4:** Cargo loading strategies for PELNs.

Category	Method	Principle	Cargo Type	Efficiency	Cargo Integrity	Ref.
Passive loading	Co-incubation	Concentration gradient difference	Small hydrophobic molecules	Low loading efficiency	Preserves vesicle membrane integrity	[112,120]
Active loading	Electroporation	Electric pulses form transient pores in the membrane	Nucleic acids/ proteins	High efficiency/Enables encapsulation of macromolecular cargos	Risk of membrane damage	[114,121]
	Sonication	Ultrasound forms transient pores	Small-molecule drugs/ nanoparticles/ proteins	High efficiency	Risk of membrane damage	[101,115]
	Freeze–thaw cycles	Repeated freeze–thaw induces membrane deformation	Proteins/small-molecule drugs	Low efficiency	/	[122]
	Extrusion	Membrane extrusion	Drug molecules/ nanomaterials/ nucleic acids/ proteins	Low efficiency	Risk of membrane structural damage	[115,123]
	Surfactant permeabilization	Chemical reagents interact with membrane lipids	Chemical drugs	High efficiency	/	[101]
Surface modification	Chemical conjugation	Chemical coupling introduces specific ligands	Targeting ligands	Strong targeting capability	/	[113]
	Electrostatic adsorption	Electrostatic interaction	Charged small molecules	/	Maintains vesicle membrane integrity	[117]
	Membrane hybridization	Membrane fusion with liposomes	Lipid membranes	/	/	[119]

## Data Availability

No new data were created or analyzed in this study. Data sharing is not applicable to this article.

## Supplementary Materials

The following supporting information can be downloaded at https://www.mdpi.com/article/10.3390/ijms27219866/s1.
