# Supplementary material for: Plant-Derived Exosome-like Nanoparticles: Biocompatible Vectors for Efficient Intervention of Autoimmune Diseases
Source: Int J Mol Sci. 2026 Nov 3;27(21):9866. doi: 10.3390/ijms27219866 (PMC13764212; doi:10.3390/ijms27219866)
Supplement: Supplementary file 1 [file ijms-27-09866-s001.zip › ijms-4529166-supplementary.pdf]

Supplementary Table S1. Abbreviations related to extracellular vesicles and exosome-like nanoparticles.

Abbreviation	Full term
PELNs	Plant-derived Exosome-Like Nanoparticles
PDEVs	Plant-Derived Extracellular Vesicles
PEVs	Plant Extracellular Vesicles
PDVLNs	Plant-Derived Vesicle-Like Nanoparticles
PDNVs	Plant-Derived Nanovesicles
EVs	Extracellular Vesicles
M-Exos	Mammalian Exosomes
MVBs	Multivesicular Bodies
ILVs	Intraluminal Vesicles
EXPO	Exocyst-positive Organelle
BELNs	Broccoli-derived Exosome-Like Nanoparticles
AELNs	Aloe-derived Exosome-Like Nanoparticles
CLEs	Curcuma longa-derived Exosome-Like Nanoparticles
GELNs	Ginger-derived Exosome-Like Nanoparticles
FA-GDEVs	Folic acid-modified ginger-derived exosome-like nanoparticles
GDNPs	Ginseng-derived exosome-like nanoparticles
Cc-ELNs	Coptis chinensis-derived exosome-like nanoparticles
B-ELNs	Broccoli-derived exosome-like nanoparticles
PO-ELNs	Portulaca oleracea-derived exosome-like nanoparticles
GELENs	Grape-derived exosome-like nanoparticles
A-ELNs	Atractylodes macrocephala-derived exosome-like nanoparticles
PENVs	Prune-derived exosome-like nanoparticles
MC-ELNs	Momordica charantia-derived exosome-like nanoparticles
TP-ELNs	Trifolium pratense-derived exosome-like nanoparticles
M-ELNs	Mango ginger-derived exosome-like nanoparticles
VDNPs	Viola Yedoensis Makino-derived exosome-like nanovesicles
GF-ELNs	Grapefruit-derived exosome-like nanoparticles
SELNs	Sea buckthorn-derived exosome-like nanoparticles
GENs	Garlic-derived exosome-like nanoparticles
CS-ELNs	Citrus sinensis-derived Exosome-Like Nanoparticles
LELNs	Lemon-derived exosome-like nanoparticles
HELNs	Herbal-derived Exosome-Like Nanoparticles
HML-ELNs	Hydrangea macrophylla leaf-derived Exosome-Like Nanoparticles
RELNs	Red cabbage-derived exosome-like nanoparticles
MSCs-EVs	Mesenchymal stem cell-derived extracellular vesicles
DR-ELNs/DR-EVs	Drynaria roosii-derived exosome-like Nanoparticles
